# A new species of *Zagrammosoma* Ashmead (Hymenoptera, Eulophidae) from Qinghai Province, China

**DOI:** 10.3897/zookeys.417.7464

**Published:** 2014-06-19

**Authors:** Huan-Xi Cao, John La Salle, Chao-Dong Zhu

**Affiliations:** 1 Key Laboratory of Zoological Systematics and Evolution, Institute of Zoology, Chinese Academy of Sciences, No. 1-5 Beichen West Road, Chaoyang District, Beijing, 100101, China; 2 University of Chinese Academy of Sciences, No.19A Yuquan Road, Shijingshan District, Beijing, 100049, China; 3 CSIRO Ecosystem Sciences, GPO Box 1700, Canberra, ACT 2601, Australia

**Keywords:** Eulophidae, *Zagrammosoma dulanense* sp. n., *Micrurapteryx sophorivora*, *Thermopsis lanceolata*, Qinghai

## Abstract

The new species *Zagrammosoma dulanense* Cao & Zhu, **sp. n.**, from Qinghai Province, China, is described and illustrated. All type specimens were reared from the pupae of *Micrurapteryx sophorivora* Kuznetzov & Tristan (Lepidoptera: Gracillariidae), a leafmining moth attacking the plant *Thermopsis lanceolata* R. Br. (Fabaceae). A key to the three known Asian species of *Zagrammosoma* is provided. All specimens are deposited in the Insect Collection, the Institute of Zoology, Chinese Academy of Sciences, Beijing, China.

## Introduction

The genus *Zagrammosoma* Ashmead is one of several small genera of the Eulophidae tribe Cirrospilini (Hymenoptera: Chalcidoidea). The members of this genus mainly attack leafminers in the orders Lepidoptera and Diptera (La Salle 1989), occasionally Coleoptera (Peck 1951) and Hymenoptera (Ubaidillah et al. 2000). They are predominantly New World in distribution. Noyes (2013) listed 16 species in this genus, with 12 of them known from the Americas (Gordh 1978, La Salle 1989, Noyes 2013). Of the Old World species, *Zagrammosoma crowei* (Kerrich) was known from East Africa and Reunion (Noyes 2013), *Zagrammosoma talitzkii* (Bouček) from Italy and Eastern Europe through to Russia, Kazakhstan and Turkmenistan (Noyes 2013, Radeghieri et al. 2002), and *Zagrammosoma latilineatum* Ubaidillah from Australia and Indonesia (Ubaidillah et al. 2000).

Noyes (2013) also included *Zagrammosoma variegatum* (Masi) in *Zagrammosoma*. Several authors discussed differences between *Zagrammosoma* and *Cirrospilus*, which are clearly closely related (Gates 2000, Gordh 1978, Hansson and La Salle 1996, La Salle 1989, Ubaidillah et al. 2000, Ubaidillah et al. 2003, Zhu et al. 2002). For the most part these authors have placed *variegatum* Masi in the genus *Cirrospilus* as *Cirrospilus variegatus* (Masi), and this paper follows that classification. It should be noted that a species of *Cirrospilus* determined as *Cirrospilus* nr *variegatus* was included in a molecular analysis of Eulophidae relationships (Gauthier et al. 2000), and it consistently clustered with *Cirrospilus* rather than *Zagrammosoma*. This is despite the fact that a morphological analysis of Cirrospilini relationships (Ubaidillah et al. 2003) found that a relationship between *Zagrammosoma* and *Cirrospilus variegatus* and the closely related *Cirrospilus afer* (Silvestri) was supported by at least one morphological character. Until further work provides a better understanding of these relationships, this paper follows the definition of *Zagrammosoma* as used or discussed by Schauff et al. (1997), Zhu et al. (2002) and Ubaidillah et al. (2003), which excludes *Cirrospilus variegatus*.

During a recent field trip to Kunlun Mountains, Qinghai Province, many specimens of a parasitoid species were reared from the pupae of *Micrurapteryx sophorivora* (Lepidoptera: Gracillariidae), a leafminer attacking *Thermopsis lanceolata* R. Br. (Fabaceae). These specimens are described and illustrated here as a new species of the genus *Zagrammosoma*, after comparing them with descriptions (Kerrich 1969, La Salle 1989, La Salle 1992, Ubaidillah et al. 2000, Yefremova 1995a, b), or specimens deposited in Natural History Museum (NHM), London, UK and those in the Insect Collection, the Institute of Zoology, Chinese Academy of Sciences (IZCAS), Beijing, China.

A key to the known Asian species of *Zagrammosoma* based on females is also provided.

## Material and methods

Host plants were collected in Qinghai Province, China in late July 2013 and moved to the lab in Beijing to rear specimens of the leafminer moths and parasitoid wasps. Specialists in the Institute of Botany, Chinese Academy of Sciences, identified the plant. All parasitoid specimens were collected and stored in 95% alcohol. Then specimens were mounted on cards for morphological studies and deposited in the Insect Collection, the IZCAS, Beijing, China.

Habitus and head pictures were recorded with a NIKON D7000 digital camera connected to a NIKON SMZ 1500 stereomicroscope. Pictures of appendages (forewings, antennae and legs) were taken by a CANON 550D digital camera connected to a LEICA DM-2500 microscope. All pictures above were stacked with Helicon Focus software.

Morphological terminology and abbreviations follow Gibson (1997). Abbreviations are: SMV, submarginal vein; MV, marginal vein; PMV, postmarginal vein; STV, stigmal vein; Gtn, gastral tergites; POL, postocellar length; OOL, ocular-ocellar length. Absolute measurements in millimeters (mm) were used for the body and forewing length. For all other dimensions, relative measurements were used.

Acronyms in this text are as follows: IZCAS, the Institute of Zoology, Chinese Academy of Sciences, Beijing, China; MZBI, Museum Zoologicum Bogoriense, Bogor, Java, Indonesia; NMPC, National Museum (National History), Prague, Czech Republic.

Unless indicated otherwise, all examined specimens are deposited in the Insect Collection, the IZCAS.

## Systematics

### Genus Zagrammosoma

Taxon classificationAnimaliaHymenopteraEulophidae

Ashmead, 1904

Hippocephalus Ashmead, 1888: viii. Type species: *Hippocephalus multilineatus* Ashmead; by monotypy; preoccupied by *Hippocephalus* Swainson, 1839 in fishes.
Zagrammosoma Ashmead, 1904: 354, 393. Replacement name for *Hippocephalus* Ashmead (not Swainson).
Zagrammatosoma Schulz, 1906: 142. Unjustified emendation.
Mirzagrammosoma Girault, 1915: 279. Type species: *Mirzagrammosoma lineaticeps* Girault; by monotypy; synonymized by La Salle 1989: 230, 232.

#### Diagnosis.

Vertex vaulted and extending above level of compound eyes; funicle 2-segmented; pronotum elongate; notaulus curved and extending to anterior half of axilla; axilla strongly advanced, typically elongate, mostly anterior to scutellum; mesoscutum elongate, longer than scutellum; scutellum with 2 pairs setae, and 2 parallel submedian grooves which are often difficult to discern due to color pattern; forewing often with fuscate areas; propodeum without plicae, but with remnants of a median carina; color at least partly yellow, often with striking patterns, not metallic.

#### Biology.

The biology of *Zagrammosoma* has been poorly studied, but its taxonomic host range seems to be quite wide but within a narrow ecological niche. Species are ectoparasitoids, mostly of the larvae or pupae of leafminers from several insect orders, including Lepidoptera and Diptera (La Salle 1989), occasionally Coleoptera (Peck 1951) and Hymenoptera (Ubaidillah et al. 2000), and in total 15 families in the above four orders (Noyes 2013).

### Checklist of known species of *Zagrammosoma* in Asia

#### 1. Zagrammosoma latilineatum

Taxon classificationAnimaliaHymenopteraEulophidae

Ubaidillah, 2000

Zagrammosoma latilineatum Ubaidillah, 2000: 223–225, *in*Ubaidillah et al. (2000). Holotype ♀, Indonesia: West Java, Bandung, Pangalengan (MZBI).

##### Distribution.

Australia; Indonesia.

##### Hosts.

DIPTERA **Agromyzidae:** *Liriomyza huidobrensis*. (Ubaidillah et al. 2000)

#### 2. Zagrammosoma talitzkii

Taxon classificationAnimaliaHymenopteraEulophidae

(Bouček, 1961)

Cirrospilus (Zagrammosoma) talitzkii Bouček, 1961: 18-19, 27-28. Holotype ♂, Moldova: Kishinev (NMPC).
Cirrospilus talitzkii Bouček, 1961: Kerrich 1969: 196.
Zagrammosoma talitzkii (Bouček, 1961): Yefremova 1995b: 50-51.

##### Distribution.

Bulgaria; Kazakhstan; Iran; Italy; Moldova; Russia; Turkmenistan; Ukraine; Yugoslavia (Federal Republic); China: Xinjiang.

##### Hosts.

LEPIDOPTERA **Bucculatricidae:** *Bucculatrix crataegi*; **Gracillariidae:** *Cameraria ohridella*, *Parornix persicella*, *Phyllonorycter connexella*, *Phyllonorycter corylifoliella*, *Phyllonorycter malella*, *Phyllonorycter saliciphaga*, *Phyllonorycter sorbi*, *Phyllonorycter spinicolella*; **Heliozelidae:** *Holocacista rivillei*; **Lyonetiidae:** *Leucoptera malifoliella*, *Leucoptera sinuella*; DIPTERA **Agromyzidae:** *Liriomyza pseudopygmina*. (Noyes 2013, Radeghieri et al. 2002, Yefremova 1995b)

#### Zagrammosoma dulanense

Taxon classificationAnimaliaHymenopteraEulophidae

Cao & Zhu sp. n.

http://zoobank.org/693F11F7-D759-4029-A81A-9FC113FA14DC

Figs 1 – 3

##### Material examined.

Holotype 1♀; Paratypes: 8♀, 4♂. China: Qinghai Province, Dulan County, 2458 Km milestone locality on G109 national highway (35°53.782'N, 97°47.106'E), 3060m; Host moth: *Micrurapteryx sophorivora* (Lepidoptera: Gracillariidae); Host plant: *Thermopsis lanceolata* (Fabaceae); 29.VII. 2013, coll. Huan-Xi CAO.

##### Diagnosis.

**Figure 1.**
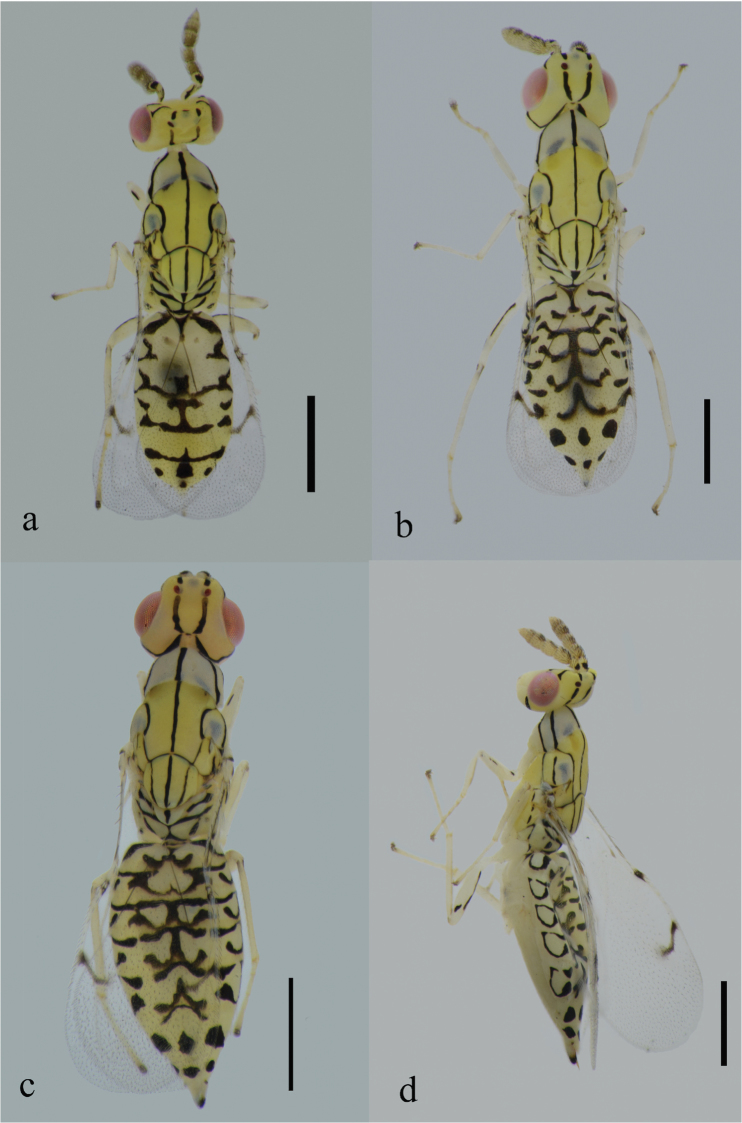
Habitus of *Zagrammosoma dulanense* Cao & Zhu sp. n.: **a** body in dorsal view (♂) **b** body in dorsal view (♀) **c** body in dorsal view (♀) **d** body in lateral view (♀). Scale bar: 0.5 mm.

Antenna (Fig. 3d) with fuscate setae on flagellum and pedicel in dorsal view. Mesosoma (Figs 1a-d) yellow with black patterns and markings except pronotum and lateral lobes of mesoscutum pale yellow. A narrow median black stripe extends the whole length of mesosoma excluding neck. Forewing (Fig. 3e) hyaline with large bare areas and fuscate parastigma and stigmal vein in contrast to light colored marginal and postmarginal veins. Stigma long and slightly curved, with uncus near its apex. This new species can be distinguished from the other Asian species by the black marking pattern on the gastral tergites (Figs 1a-d), on which there are three black spots on Gt_6_ and Gt_7_ respectively in female, and markings on Gt_7_ are reduced into one median black spot in male.

The key provided here will differentiate *Zagrammosoma dulanense* from other Asian species; it can be distinguished from any New World species with even vaguely similar body coloration by the following characters of the forewing: surface of forewing (Fig. 3e) completely hyaline and without dark markings except for a very small patch near stigma, stigmal vein (particularly at base) and parastigma bordering marginal vein with dark areas in contrast to light colored marginal and postmarginal veins.

##### Description.

**Female.** Body length 2.0–3.1 mm.

**Color.** Body yellow with black stripes and spots (Fig. 1b–d).

**Figure 2.**
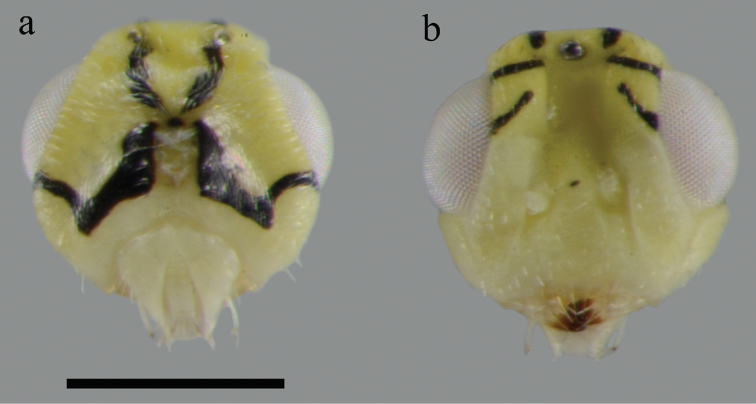
Head (♀): **a** head in posterior view **b** head in anterior view. Scale bar: 0.2 mm.

**Figure 3.**
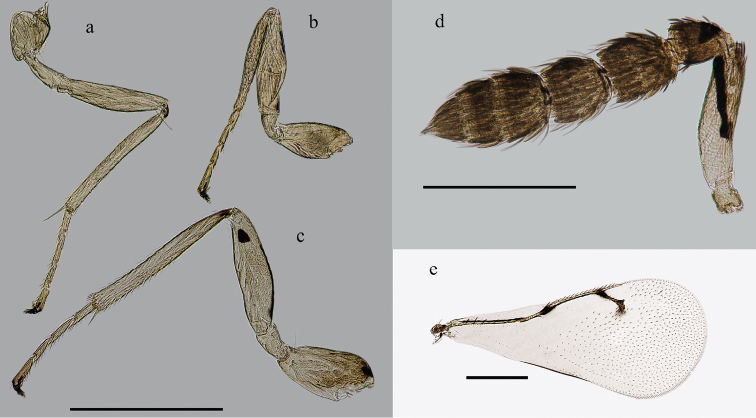
Appendages (♀): **a** mid leg **b** fore leg **c** hind leg **d** antenna **e** forewing. Scale bar: 0.5 mm.

Frons yellow with two sets of short black stripes laterally extending from below the flange of vertex to upper lateral eye margin (Fig. 2b). Occipital foramen with a black spot. Two pairs of dark stripes diverging from occipital foramen, one pair diverging upward to the anterior ocellus, truncated into two pieces by posterior ocelli, and the other pair proceeding ventrally and curving antero-dorsally to meet ventral eye margin (Fig. 2a). Antenna yellow, scape with a oblique black stripe on dorsal surface, pedicel with a black spot basally on dorsal surface (Fig. 3d).

Pronotal collar and lateral lobe of mesoscutum pale yellow. A black stripe extending from base of pronotum to apex of scutellum medially, forming a reverse triangle on dorsellum. Two enlarged dark spots anterolaterally on mesoscutum, which are partially visible through the pronotum which overlaps them. Mesoscutum with dark stripe bordering axilla. Two parallel black stripes on lateral lobe of mesoscutum in lateral view. Acropleuron and upper mesepimeron with a dark stripe respectively; sometimes the one on upper mesepimeron truncated into two short parts. The border of metapleuron covered by dark stripe. Dark stripes along notauli and scuto-scutellar suture. Two dorsolateral dark stripes on scutellum, slightly diverging anteriorly and fused posteriorly. Lateral panel of metanotum with short black curved stripe. Propodeum with a dark stripe along the anterior margin and a W-shaped dark marking below it; the median line of “W” bold basally and apically, reaching the anterior margin.

Metasoma yellow with black coloration pattern (Fig. 1b–d). Black markings on Gt_1_-Gt_5_ extend postero-laterally and develop the forms of “W” with straight or arched stripes below them; sometimes these stripes absent. Markings on Gt_6_ and Gt_7_ reduced to three black spots respectively.

Wings hyaline; vein pale yellow with the exception of fuscate parastigma and STV; SMV with a black line on it; humeral plate and tegula with one dorsal dark spot respectively. Legs (Fig. 3a–c) yellow; middle and hind coxae with dark dorsal spot; hind femur with black stripe on dorsal surface extending from base approximately three quarters the length of femur, and a black spot near apex of femur.

**Head:** Head (Fig. 2a–b) nearly quadrate in anterior view, with numerous short white setae. Mandible brown apically. Vertex with several scattered black setae dorsally, vaulted between compound eyes. Face with small and scattered white setae, difficult to see due to the coloration pattern. Eyes bare. Toruli placed slightly above the level of lower eye margin. Relative measurements: POL 12, OOL 8.

**Antenna:** Antenna (Fig. 3d) with two anelli, two funicular segments and a 3-segmented club. The first funicle slightly longer than the second segment with ratio of length 11/9; the first funicle slightly longer than its width, the second segment as long as wide. Ratios of the length of three club segments: 8/6/6; the second club segment more transverse. All segments excluding scape and anelli setose with several fuscate setae in dorsal view. Longitudinal sensilla present on all funicular and club segments.

**Mesosoma:** Mesosoma (Fig. 1b–d) with fine reticulate sculpture dorsally and laterally, and several short white setae scattered on the midlobe of mesoscutum. Notaulus curving to meet axilla. Scutellum with 2 pairs of long white setae. Lateral panel of metascutum smooth; dorsellum large, triangular, the tip pointed. Propodeum with fine reticulate sculpture, callus with some white erect setae, spiracle small and round.

**Metasoma:** Metasoma with fine reticulate sculpture, pointed apically. Short white setae uniformly distributed on metasoma. Metasoma subequal in length with mesosoma, and slightly wider. Length ratio of metasoma/mesosoma: 100/105; width ratio of metasoma/mesosoma: 60/55. Petiole very short and transverse, not visible in dorsal view. Three dark cercal setae present, which are slightly different in length. Tips of ovipositor sheath visible dorsally.

**Legs.** Legs (Figs 3a–c) with numerous setae on tibiae and tarsi. Tibial spur on each leg shorter than basitarsus, particularly on fore leg.

**Forewing:** Forewing (Fig. 3e) length 1.5–1.8 mm, with large bare areas extending from base of basal cell to STV except for several admarginal setae on ventral surface of wing below MV. Costal cell with several pale setae on its dorsal surface. SMV with 5–10 dorsal setae, MV with a row of black setae. SMV longer than MV; PMV shorter than STV. Relative lengths of veins: SMV/MV/PMV/STV: 60/40/10/19. Several black setae on STV, stigma long and slightly curved, with uncus near its apex. The triangular area on wing surface between PMV and STV bare. Speculum open. Basal setal line absent. Cubital setal line absent at base, and its three or less setae extend into speculum. Subcubital line very close to the margin of forewing.

**Variation:** Apart from different body sizes of specimens, the main variation is in the color pattern, particularly on propodeum and metasoma. On propodeum, the lateral arms of W-shaped markings may not connect to the posterior transverse marginal stripe. On metasoma, variations include changes in thickness of stripes and whether the black transverse stripes below the W-shaped dark markings exist, and if exist, whether connecting to the W-shaped dark markings. Occasionally, there is a small dark spot between the toruli.

**Male:** Smaller than female. Body length 1.8–2.3 mm. Forewing length 1.3–1.6 mm. Antenna with numerous setae on flagellar segments, more setose than that of female. The significant difference between female and male lies in the pattern of stripes on metasomal tergites, which are often paler and less extensive than that of female (Fig. 1a). Gt_7_ shorter than other metasomal tergites by comparison, and markings on it reduced to one black spot. Genitalia protruding in dorsal view.

**Biology:** The new species has been reared from the pupae of *Micrurapteryx sophorivora* Kuznetzov & Tristan (Lepidoptera: Gracillariidae) on *Thermopsis lanceolata* R. Brown (Fabaceae).

Gençer and Seven (2005) reared 6 eulophid species (*Baryscapus nigroviolaceus*, *Cirrospilus pictus*, *Necremnus croton*, *Neochrysocharis arvensis*, *Neochrysocharis formosa*, *Pnigalio* sp.) and one pteromalid (*Pteromalus* sp.) from *Micrurapteryx sophorivora* mining *Robinia pseudoacacia* (Fabaceae) in Turkey.

The host plant is toxic and can cause livestock poisoning, but has also been used as a Chinese medicine plant (Zhu and Kirkbride 2006). Adults emerged from the host moth throughout August 2013, with fewer female specimens reared in September, while three males were collected in October. To our surprise, another female was captured from the dry host plant kept in the lab until mid May, 2014.

##### Distribution.

China: Qinghai Province.

##### Etymology.

This species is named for the locality in Dulan County, Qinghai Province, China, where the host plant, moth and type specimens were collected.

### Key to the known Asian species of *Zagrammosoma* based on females

Note that some species of *Cirrospilus*, e.g. *Cirrospilus variegatus*, appears very close to *Zagrammosoma*, and even have a slightly to distinctly vaulted vertex. These species of *Cirrospilus* can be distinguished by having the notaulus straight posteriorly and extending to the scuto-scutellar suture.

**Table:** 

1	Propodeum almost entirely dark; scutellum without dorsolateral stripes; median dark stripe on mesoscutum wide posteriorly, over half the width of scuto-scutellar margin	*Zagrammosoma latilineatum*
–	Propodeum almost entirely yellow but with dark stripes or markings; scutellum with dorsolateral stripes; median dark stripe on mesoscutum narrow, less than half the width of scuto-scutellar margin	2
2	Pronotum and lateral lobes of mesoscutum yellow; posterior margin of pronotum without dark or brown markings; speculum small with setae on dorsal surface of wing surface extending to line of admarginal setae on ventral surface; metasoma with short dark setae; three black spots on Gt_6_ in the form of a triangle; Gt_7_ with one small black spot	*Zagrammosoma talitzkii*
–	Pronotum and lateral lobes of mesoscutum pale yellow; posterior margin of pronotum with enlarged dorsolateral dark or brown spots; speculum large with setae on dorsal surface of wing clearly separated from line of admarginal setae on ventral surface; metasoma with short white setae; three black spots on Gt_6_ in a straight line; Gt_7_ with three black spots	*Zagrammosoma dulanense* sp. n.

## Supplementary Material

XML Treatment for Zagrammosoma

XML Treatment for Zagrammosoma latilineatum

XML Treatment for Zagrammosoma talitzkii

XML Treatment for Zagrammosoma dulanense
